# Potential surrogate plants for use in semi-field pesticide risk assessment with *Megachile rotundata*

**DOI:** 10.7717/peerj.6278

**Published:** 2019-01-18

**Authors:** Andrew J. Frewin, Angela E. Gradish, Graham R. Ansell, Cynthia D. Scott-Dupree

**Affiliations:** School of Environmental Sciences, University of Guelph, Guelph, Ontario, Canada

**Keywords:** Semi-field, Risk assessment, Non-apis, Solitary bee

## Abstract

**Background:**

Current regulatory pesticide risk assessments for bees are based primarily on the honey bee (*Apis mellifera*) and may not always be protective of solitary bees. To incorporate solitary bees into the risk assessment process, standardized methods to assess the hazard of pesticides under semi-field (Tier II) conditions will be needed. We conducted a series of experiments over 2 years to assess potential surrogate plants and adult release rates for use in semi-field experiments with the alfalfa leafcutting bee (ALB, *Megachile rotundata*).

**Methods:**

We compared ALB foraging activity and reproduction on 12 m^2^ plots of flowering alfalfa (*Medicago sativa*) and buckwheat (*Fagopyrum esculentum*) at low (10♀/20♂) and high (20♀/40♂) adult release rates. The following year, we assessed the same endpoints on plots of purple tansy (*Phacelia tanacetifolia*) at a release rate of 10♀/15♂.

**Results:**

Although ALB foraging activity was high on buckwheat plots, fewer adults were produced compared to alfalfa plots. On alfalfa, there were no differences in foraging activity, nesting, or reproduction between the low and high release rates. ALB readily foraged from purple tansy flowers, but females avoided purple tansy leaves for leaf cell construction.

**Discussion:**

Our study suggests that buckwheat alone cannot support ALB during semi-field studies on small plots. For alfalfa, we recommend a maximum release rate of 10♀/20♂ in 12 m^2^ plots. Further study of higher ALB release rates on purple tansy is warranted. A mixed planting of purple tansy and a plant suitable for leaf piece collection (e.g., buckwheat) may provide favorable conditions for ALB activity and reproduction during semi-field testing.

## Introduction

Bees are key animal pollinators of many wild and agricultural plants (Potts et al., 2010; Kleijn et al., 2015). To date, bee-related research efforts have been largely focused on honey bees (*Apis mellifera* L.) and, more recently, bumble bees (*Bombus* spp.), with far less emphasis placed on solitary bees. This disproportionate focus has occurred even though approximately 85% of bee species are solitary (Michener, 2000), and solitary bees are important natural and managed pollinators in several agroecosystems (Kleijn et al., 2015).

Bee population and species declines have recently been documented globally, and the potential role of pesticides in these losses has become the subject of intense scientific, public, and regulatory scrutiny and debate (Potts et al., 2010; Cameron et al., 2011; Rundlof et al., 2015; Woodcock et al., 2016). These concerns have led to a re-evaluation of the regulatory pesticide risk assessment process for bees in in North America and the European Union (EFSA, 2012; EFSA, 2013; USEPA, PMRA & CDPR, 2012; USEPA, PMRA & CDPR, 2014). A risk assessment for bees is required for pesticide registration and re-registration, and historically, these assessments have primarily relied on the honey bee as a surrogate species to estimate the risk of pesticide exposure for all bees. However, because of their radically different life history and behavior, solitary bees may differ in their susceptibility to pesticides compared to social bees (Scott-Dupree, Conroy & Harris, 2009; Brittain & Potts, 2011; Arena & Sgolastra, 2014), and thus, current pesticide risk assessment frameworks may not be protective of solitary bees (OECD, 2010; EFSA, 2012; EFSA, 2013; USEPA, PMRA & CDPR, 2012; USEPA, PMRA & CDPR, 2014). Because of these differences, combined with the lack of pesticide toxicity and exposure data for solitary bees, regulatory agencies are expected to request solitary bee data to support pesticide registrations (OECD, 2010; EFSA, 2012; EFSA, 2013; USEPA, PMRA & CDPR, 2012; USEPA, PMRA & CDPR, 2014).

Regulatory pesticide risk assessment for bees proceeds through three tiers of testing using standardized and validated methods (OECD, 2010; USEPA, 2016; Health Canada Pest Management Regulatory Agency, 2017). Tier 1, laboratory studies are designed to screen potentially harmful pesticides under worst-case conditions where individual bees are exposed to a known amount of a pesticide. The objective of tier 1 studies is typically to determine critical values (e.g., lethal median dose, LD_50_) that can be used to compare the relative toxicity of pesticides. In tier 2, semi-field experiments, bees are confined to a pesticide-treated crop in the field and impacts on colony reproduction, and behaviour are assessed. Finally, in tier 3 field experiments, bees are allowed to free forage in close proximity to a treated crop and impacts on colony reproduction, and behaviour are assessed. Because honey bees currently serve as the surrogate bee species for regulatory pesticide risk assessments, standardized and validated risk assessment methods have been established for them at all three tiers (Alix et al., 2014; Lee-Steere & Steeger, 2014; OECD, 1998a; OECD, 1998b; OECD, 2007; USEPA, 2016). However, because of their biological and behavioral differences, these methods cannot be directly applied to solitary bees. Therefore, to include solitary bees in the pesticide risk assessment process, methods at all three tiers specific to solitary bees are needed.

In this paper, we summarize a series of studies we conducted to contribute towards the development of a semi-field method for use in the pesticide risk assessment process for the alfalfa leafcutting bee (ALB, *Megachile rotundata* F.). ALB is a palearctic species that established in North America in the early 20th century and is now a managed pollinator of alfalfa and other crops (Pitts-Singer & Cane, 2011). Because its biology and life history are well understood, and it is commercially available with an established rearing technique, the ALB would be an ideal surrogate species for solitary bee pesticide risk assessment in North America.

We focused on two basic elements of the semi-field experimental design for the ALB. Our first objective was to identify a suitable surrogate plant(s) for semi-field studies with ALB. Semi-field studies involve confining bees to a pesticide-treated, flowering surrogate plant in the field and assessing lethal and sub-lethal effects. Thus, the surrogate plant must elicit high foraging activity by adults to ensure exposure to the test pesticide, and it must produce nutritious nectar and pollen to support adults and developing brood during testing (Gradish et al., 2016). For ALB, the surrogate plant foliage also must be suitable for collection of leaf pieces for construction of brood cells by females. Based on these criteria, we compared ALB foraging activity, nesting behavior, reproduction, and offspring development on alfalfa (*Medicago sativa*), buckwheat (*Fagopyrum esculentum*), and purple tansy (*Phacelia tanacetifolia*), known forage plants of ALB (Horne, 1995a; Pitts-Singer & Bosch, 2010; Artz & Pitts-Singer, 2015), under semi-field conditions. Our second objective was to determine a male and female release rate that optimizes ALB reproduction under our semi-field conditions. The adult release rate must ensure that reproduction and female nesting is high enough to detect potential sub-lethal effects of the test pesticide. However, after a certain threshold, increasing release rates may impair female nest establishment and reproductive success (Pitts-Singer & Bosch, 2010) or increase harassment of females by males (Rossi, Nonacs & Pitts-Singer, 2010), which can ultimately impede reproduction and fecundity. Thus, the optimal ALB adult release rate for a given surrogate plant is one that maximizes nesting activity and reproduction without greatly increasing competition. We quantified ALB nesting behaviour and reproduction under different adult release rates on alfalfa and buckwheat. Finally, our third objective was to generally assess the suitability of small enclosures (Gradish et al., 2016) for semi-field studies with ALB.

## Methods

### Alfalfa leafcutting bees

ALB were purchased from NorthStar Seeds Ltd. (Neepawa, MB) in the winter of 2015 and 2016 as diapausing prepupae and stored at 4 °C until use. All bees were used within 1 y of purchase. Development was induced by placing prepupae in 2 L ventilated plastic containers in an incubator at 29 ± 2 °C, 60% RH, 12:12 h light:dark cycle (Richards & Whitfield, 1988). Adult emergence was monitored daily, and only bees less than 48 h old were used in experiments.

The evening before all experiments, the thorax of each female ALB to be released in each semi-field plot was uniquely marked with a Sharpie^®^ Poster-Paint water-based paint marker. Identification marks consisted of either a single large dot, two small dots (same or different colors), or a stripe, allowing for 20 unique combinations. Females were held at 16 °C for no longer than 1 h to facilitate the application of identification marks, after which they were placed in storage containers with access to 20% w:v sugar-water solution and held at 24 ± 2 °C. Male bees were held separately from females under identical conditions. Males were not marked.

### Study sites

The study was conducted at two sites in southern Ontario, Canada in 2016 and 2017. The first site was located approximately 8 km south of Tillsonburg, ON. At this site 6 ha of buckwheat (var. common) was seeded at a rate of 23 kg/ha on May 19, 2016 and 30 kg/ha on June 8, 2017. At this same site, a plot (375 m^2^) of purple tansy was seeded on June 1, 2016 and June 19, 2017, at a rate of 33.6 kg/ha. Approximately one week prior to seeding, Roundup WeatherMAX^®^ (Monsanto Canada, Winnipeg, Canada) was applied to the field at 3.75 L/ha. No pesticides were applied to the field after seeding.

The second site, located approximately 3 km west of Waterford, ON, Canada, was a 5 ha established alfalfa pasture maintained by the landowner for hay production. No pesticides were applied to this field in 2016 or 2017.

### Surrogate plant and adult release rate experiments

**Figure 1:**
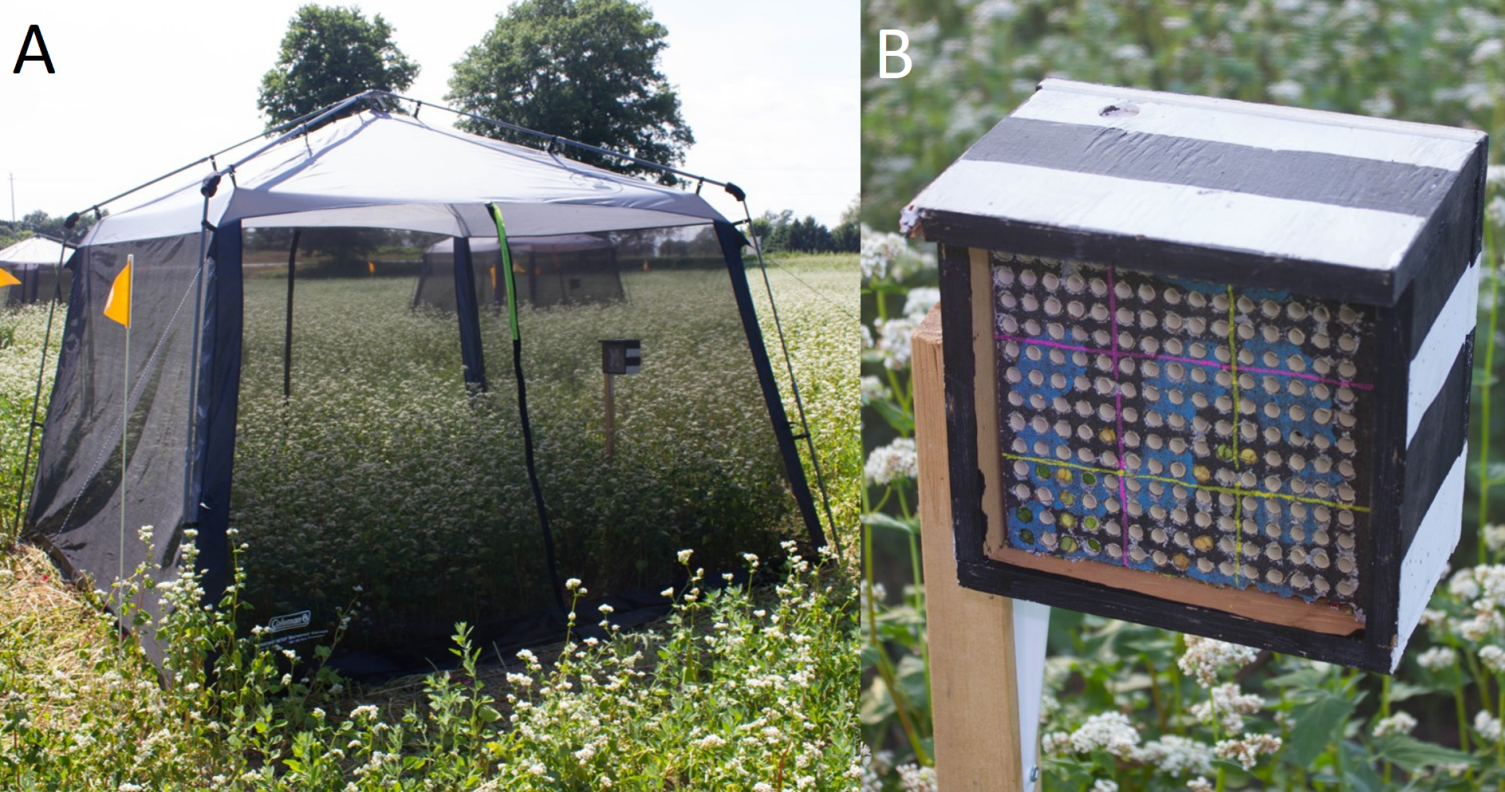
Field enclosure with nest box (A) and a close up of a nest box (B) used for semi-field experiments with *Megachile rotundata* in this study.

Experiments took place in buckwheat and alfalfa in July 2016 and purple tansy in August 2017 (purple tansy failed to germinate in 2016, presumably due to exceptionally droughty conditions that year following seeding). Experimental plots (12 m^2^) were established in the buckwheat (*n* = 8) and purple tansy (*n* = 4) when the plants were between 2nd and 4th leaf stage. Fewer purple tansy plots were established because plant germination was poor in some areas, limiting the space available for plots. However, the plants where the plots were established appeared healthy. All plots were established at least 3 m apart (1 m apart in purple tansy because of the limited space) in areas where plants were of similar density, growth stage, and health. Plots were scouted weekly and hand-weeded as needed. A screened enclosure (3.35 ×  3.35 ×  2.29 m, Instant Screen House^^®^^; Coleman Canada Inc., Brampton, Ontario, Canada) was placed over each buckwheat and purple tansy plot when the plants reached 30% bloom by visual estimate (Fig. 1A). Screened enclosures were installed on the alfalfa plots (*n* = 8) when the plants were at the early flower stage, defined as having 1 node with at least 1 open flower (Undersander et al., 2011). Enclosures were set up according to manufacturer instructions; however, manufacturer tent pegs were replaced with 20 cm ABS plastic arrowhead tent pegs. Wooden strapping was placed on the flaps at the base of each side and nailed into the ground, preventing bees from escaping and/or pests from entering the tents. An ALB nest box was then installed in each enclosure. A nest box consisted of a plywood box (10 × 10 × 18 cm) attached to a 1.4 m wooden stake. The plywood box was painted with black and white stripes and housed a commercial Styrofoam^®^ leafcutter nest block (NorthStar Seeds Ltd., Neepawa, MB, Canada) with approximately 225 nest cavities. Nest box cavities were lined with chlorine-free drawing paper to facilitate removal of brood cells (Fig. 1B). In each enclosure, the nest box was placed in the corner to the right of the entrance, 1.5 m from the enclosure sides and oriented such that the nest cavity openings faced southeast.

ALB were released into the enclosures the morning after the nest boxes were placed on the plots. In the alfalfa and buckwheat, adults were released at rates of 10♀/20♂ (*n* = 4) or 20♀/40♂ (*n* = 4) and at a single rate of 10♀/15♂ on all purple tansy plots.

Observations of adult foraging behavior were conducted on sunny or mostly sunny days for up to 18 days following the release of ALB (alfalfa and buckwheat: *n* = 7; purple tansy: *n* = 4; Table S1). On each day, observations were repeated three times between 9:15 am and 3:30 pm. The order in which plots were observed was randomized between and within days. Observations were conducted as follows: The observer entered the enclosure on the side closest to the nest box and waited 1 min for the bees to acclimate to their presence. Using a hand tally, the number of active adults was counted over a 15 s visual sweep of the enclosure. An active adult was defined as a male or female ALB observed flying and/or foraging in or just above the plant canopy. This measurement was repeated twice. Next, the number of females resting in the nest box was recorded. A female was considered resting if it was blocking the entrance to a nest cavity with its head facing outward for at least 10 s. After this, the nest box was observed for 10 min, and the number of provisioning trips and identity of the female that made each provisioning trip were recorded. A provisioning trip was counted when any female returned to the nest with a leaf piece and/or pollen; therefore, in some cases multiple trips were recorded for the same female(s). After the nest box observation was complete, two more active adult counts were made (a total of four per observation). In 2016, temperature was recorded using handheld digital thermometer (Fisherbrand™ CON4095, Fisher Scientific) at the time of observation, while in 2017, temperature was recorded using portable data loggers (HOBO^®^ Pro v2 ext temp/RH, U23-002, ONSET).

After the final observation period, nest boxes were moved to the University of Guelph, placed individually in BugDorm-2120F Insect Rearing Tents (MegaView Science, Taichung, Taiwan), and maintained in a greenhouse. All marked females that emerged from these nest boxes were collected.

Two weeks after the last emergence of second generation offspring, nest boxes were deconstructed. Brood cells were removed from the cavities, individually weighed and placed in 128-well bioassay trays (C-D International, Pitman, NJ), and stored at 4 °C. After 5 months, brood cells were moved to an incubator at 29 ± 2 °C, 60% RH, 12:12 h light:dark cycle to induce development. All emerged adults were collected, dried at 65 °C for 72 h, individually weighed (Sartorius Extend Model ED124S balance, Sartorius AG, Goettingen, Germany), and sexed. All remaining non-viable cells were dissected and classified according to their contents (a pollen ball, dead larva, or other non-viable content, which included cells with loose pollen, stacked leaves, or cells infected with a fungal pathogen).

### Data analysis

All data analyses were performed at a significance level of *α* = 0.05 in R v3.5.1 (R Core Team, 2018). Data collected from the alfalfa and buckwheat plots were analyzed together; however, because of differences in the timing of experiments, release rates, and number and frequency of observations, data from purple tansy plots were analyzed separately and included only the effects of temperature and days after release.

For buckwheat and alfalfa plots, the effects of surrogate plant, release rate, days after release, and temperature on the number of provisioning trips, active adults, and resting females were analyzed using a general estimation equation with the package ‘geepack’ (Halekoh, Højsgaard & Yan, 2016). This statistical approach was chosen because the data consisted of longitudinal counts and the analysis is considered relatively conservative. Wald’s test was used to assess significant effects, and models were validated by inspecting residuals plots following the methods outlined in Zuur et al. (2009).

The effects of surrogate plant and release rate on the number of brood cells produced, number of adults produced, and cell weights were analyzed using a linear mixed model with the package ‘nlme’ (Pinheiro et al., 2018). Adult weight data were also analyzed with a linear mixed model but included the additional effect of sex. For both models, plot was included as a random effect. AIC was used to determine the best-fit model. Models were validated by inspecting residuals plots following the protocol outlined in Zuur et al. (2009). Means were separated using Tukey’s tests.

The proportion of brood cells that contained adults, dead larvae, pollen balls, or other material were analyzed with a generalized linear model with a binominal distribution. Variance was partitioned into the fixed effects of surrogate plant, release rate, and their interaction. Significant effects were assessed with F tests.

## Results

### Alfalfa and Buckwheat

#### Nesting and foraging activity

**Figure 2:**
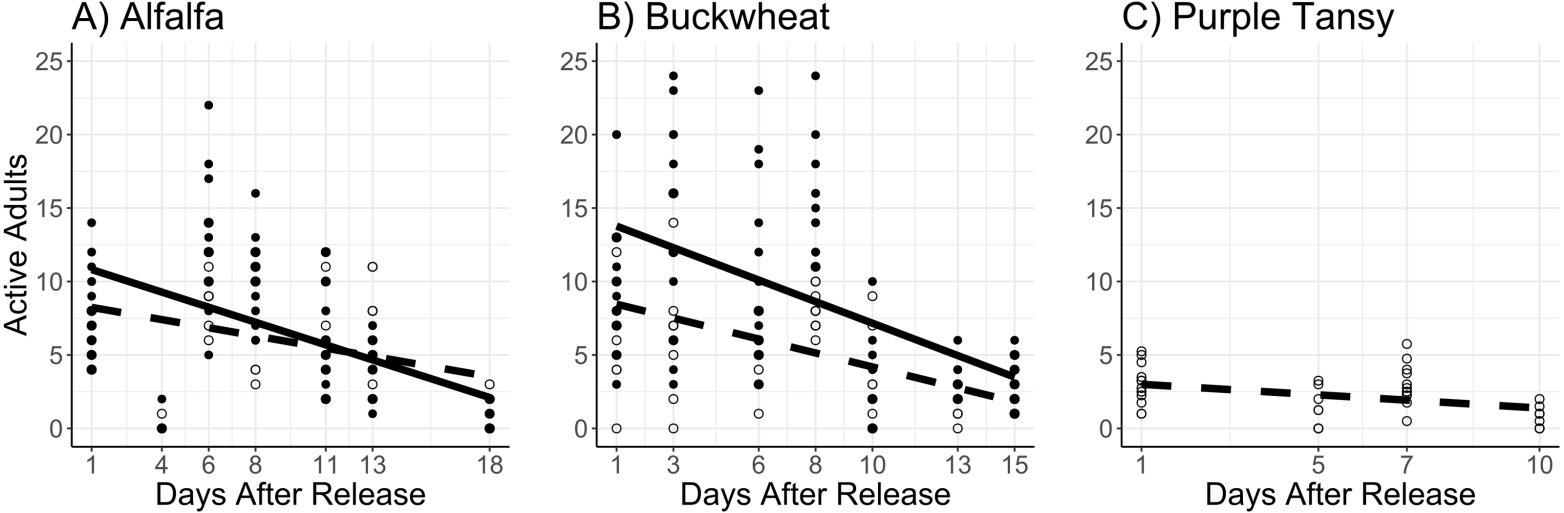
Mean number of active *Megachile rotundata* adults per observation period. Adults were released on and confined to small plots of flowering alfalfa (A), buckwheat (B), or purple tansy (C) for up to 18 days. Open circles and dashed lines represent a release rate of 10♀/20♂(A, B) or 10♀/15♂(C), and closed circles and solid lines represent a release rate of 20♀/40♂(A, B).

The interaction of surrogate plant and release rate (*χ*^2^ = 9.5; *df* = 1; *P* = 0.0020) had a significant effect on the number of active adults. More active adults were observed on buckwheat plots at the higher release rate, but there was no difference between release rates on alfalfa plots (Figs. 2A, 2B). The number of active adults also increased with temperature (*χ*^2^ = 51.6; *df* = 1; *P* < 0.005) and decreased with days after release (*χ*^2^ = 133.0; *df* = 1; *P* < 0.005) (Figs. 2A, 2B).

**Figure 3:**
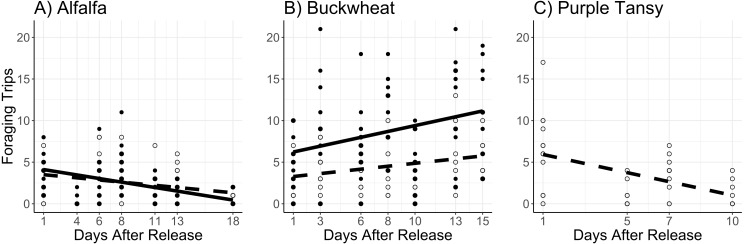
Total number of foraging trips (collection of leaf pieces and/or pollen) made by *Megachile rotundata* adults per observation period. Adults were released on and confined to small plots of flowering alfalfa (A), buckwheat (B), or purple tansy (C) for up to 18 days. Open circles and dashed lines represent a release rate of 10♀/20♂(A, B) or 10♀/15♂(C), and closed circles and solid lines represent a release rate of 20♀/40♂(A, B).

The number of foraging trips was affected by the interaction of surrogate plant and release rate (*χ*^2^ = 11.8; *df* = 1; *P* < 0.005) and the interaction of surrogate plant and days after release (*χ*^2^ = 34.5; *df* = 1; *P* < 0.005). In the alfalfa the number of foraging trips decreased with days after release and was not different between release rates (Fig. 3A). In the buckwheat plots the number of foraging trips increased over time and was greater at the high release rate (Fig. 3B). The number of foraging trips also increased with temperature in both the alfalfa and buckwheat (*χ*^2^ = 31.8; *df* = 1; *P* < 0.005).

**Figure 4:**
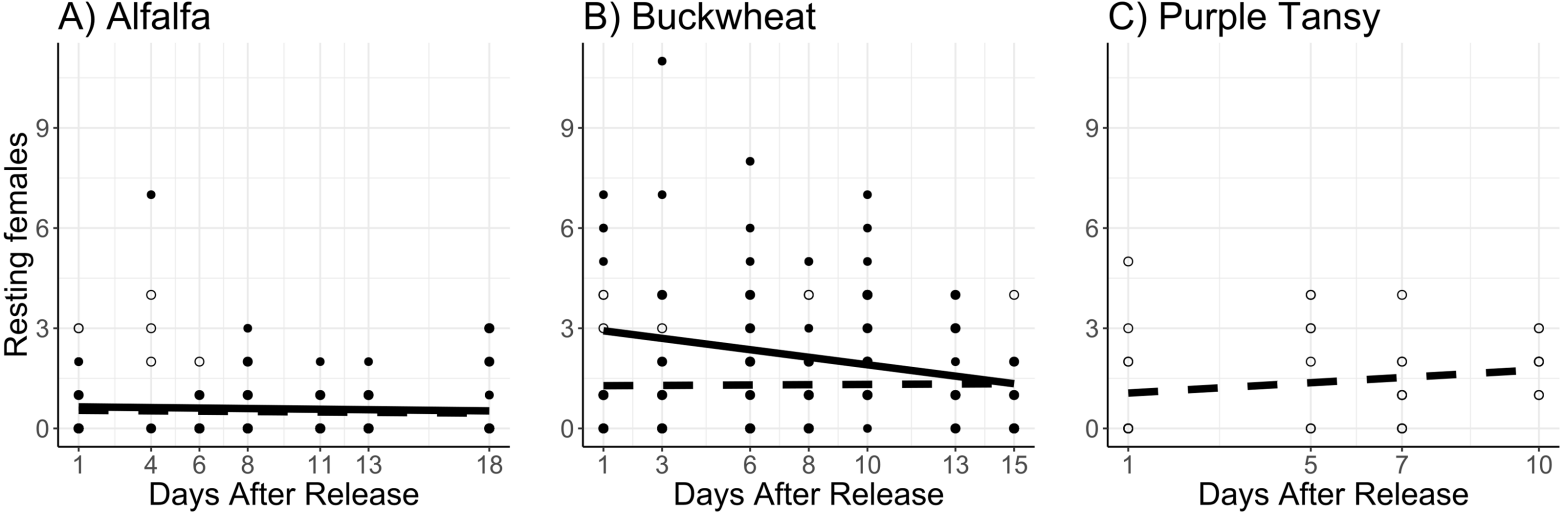
Total number of *Megachile rotundata* females resting in the nest box per observation period. Adults were released on and confined to small plots of flowering alfalfa (A), buckwheat (B), or purple tansy (C) for up to 18 days. Open circles and dashed lines represent a release rate of 10♀/20♂(A, B) or 10♀/15♂(C), and closed circles and solid lines represent a release rate of 20♀/40♂(A, B).

Surrogate plant had a significant effect on the number of resting females (*χ*^2^ = 16.9; *df* = 1; *P* < 0.005). More resting females were observed on buckwheat plots compared to alfalfa plots (Figs. 4A, 4B). Release rate also affected the number of resting females (*χ*^2^ = 51.6; *df* = 1; *P* < 0.005), as more were observed at the high release rate (Figs. 4A, 4B). The number of resting females decreased with days after release (*χ*^2^ = 4.3; *df* = 1; *P* = 0.038). The number of resting females was also affected by temperature (*χ*^2^ = 38.5; *df* = 1; *P* < 0.005), with fewer resting females observed at higher temperatures.

**Figure 5:**
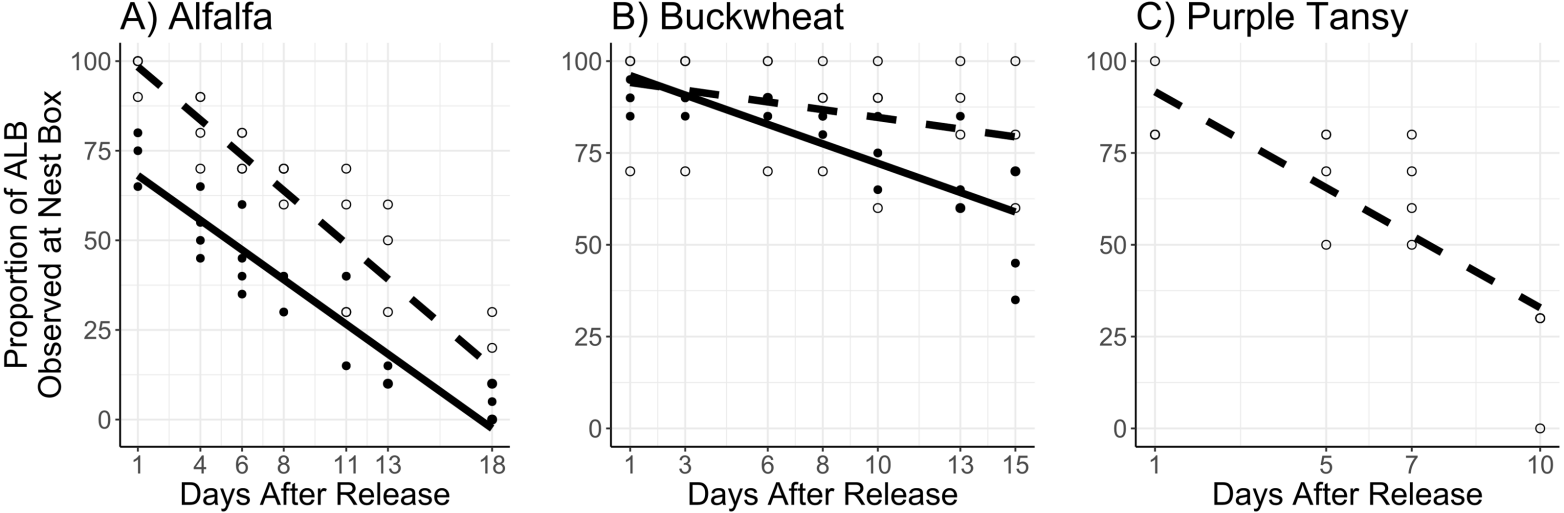
Proportion of female *Megachile rotundata* observed at the nest box per observation period. Adults were released on and confined to small plots of flowering alfalfa (A), buckwheat (B), or purple tansy (C) for up to 18 days. Open circles and dashed lines represent a release rate of 10♀/20♂(A, B) or 10♀/15♂(C), and closed circles and solid lines represent a release rate of 20♀/40♂(A, B).

The proportion of females observed at the nest box was affected by the interaction of surrogate plant and days after release (*F* = 8.78; *df* = 1; *P* = 0.003) and by release rate (*F* = 11.05; *d* = 1; *P* < 0.005). In general, a larger proportion of females were observed at the next box at the low release rate, and this effect was more apparent in the alfalfa than the buckwheat (Figs. 5A, 5B).

#### Reproduction and development

The number of brood cells produced per plot was affected by the surrogate plant/release rate interaction (*F* = 1.74; *df* = 1, 12; *P* = 0.0066). Females on buckwheat plots at the high release rate produced the most brood cells (Table 1). The remaining treatments were not significantly different from each other (Table 1).

**Table 1:** Mean (±SE) number and weight of cells and the mean number of adults produced from nests of *Megachile rotundata * confined to flowering alfalfa, buckwheat, or purple tansy plots for up to 18 days.

	Alfalfa		Buckwheat		Purple Tansy^b^
Release rate	10♀/20♂	20♀/40♂	10♀/20♂	20♀/40♂	10♀/15♂
Mean number of brood cells per plot (*n*)	42.25 ± 10.95 b^a^ (169)	35.25 ± 10.95 b (141)	30.00 ± 10.95 b (120)	94.75 ± 10.95 a (379)	41.50 ± 4.35 (166)
Mean brood cell weight (mg) (*n*)	88.21 ± 2.33 a (169)	80.00 ± 3.04 a (141)	88.69 ± 2.68 a (120)	87.65 ± 1.14 a (379)	120.55 ± 2.13 (166)
Mean number of adults produced per plot (*n*)	17.25 ± 4.18 a (69)	7.50 ± 4.18 a (30)	2.50 ± 4.18 a (10)	11.25 ± 4.18 a (45)	12.25 ± 5.12 (49)

**Notes.**

a Means followed by different letters within a row are significantly different (*α* = 0.05).

b Data collected from purple tansy plots were not included in statistical analyses; see methods for details.

Neither surrogate plant (*F* = 1.48; *df* = 1, 12; *P* = 0.2467) nor release rate (*F* = 2.54; *df* = 1, 12; *P* = 0.1372) had an effect on the weight of brood cells produced on buckwheat or alfalfa plots (Table 1).

The mean number of adults produced was affected by the plant/release rate interaction (*F* = 4.90; *df* = 1, 12; *P* = 0.047). However, poc-hoc tests indicated that none of the treatment combinations were significantly different from each other (Table 1). The highest number of adults was produced on alfalfa plots at the low release rate, whereas the fewest were produced on buckwheat plots at the low release rate.

Adult weight was affected by the interaction of surrogate plant and sex (*F* = 10.58, *df* = 1, 104; *P* = 0.0015), as well as release rate (*F* = 5.40; *df* = 1, 9; *P* = 0.045). In general individuals produced on alfalfa plots weighed more than those produced on buckwheat plots (Table 2). Females produced on alfalfa plots weighed more than males produced on alfalfa within each release rate. However, there was no difference in weight between males and females produced on buckwheat plots (Table 2).

**Table 2:** Mean (±SE) weight of *Megachile rotundata * adults. Adults were the offspring of *M. rotundata * that had been confined to plots of flowering alfalfa, buckwheat, or purple tansy plots for up to 18 days.

Forage plant	Sex	Release rate	Mean adult weight (mg)^a^
Alfalfa	M	10♀/20♀	12.88 ± 0.37 bc
	F	10♀/20♂	15.71 ± 0.51 a
	M	20♀/40♂	11.77 ± 0.54 cd
	F	20♀/40♂	14.59 ± 0.64 ab
			
Buckwheat	M	10♀/20♂	7.86 ± 0.61 e
	F	10♀/20♂	7.00 ± 1.03 de
	M	20♀/40♂	6.74 ± 0.51 e
	F	20♀/40♂	5.89 ± 0.91 e
Purple Tansy^b^	M	10♀/15♂	10.31 ± 0.72 b
	F	10♀/15♂	13.13 ± 0.87 a

**Notes.**

a Means within a column followed by different letters are significantly different (*α* = 0.05).

b Data collected from purple tansy plots were not analyzed with data from buckwheat or alfalfa plots; see methods for details.

Finally, the proportion of brood cells from which an adult emerged was affected by surrogate plant (*F* = 9.48; *df* = 1; *P* = 0.0082) and was higher in the alfalfa compared to the buckwheat (Table 3). The proportion of brood cells that contained a dead larva was also affected by surrogate plant (*F* = 48.20; *df* = 1; *P* < 0.001) and was higher in the buckwheat compared to the alfalfa. Surrogate plant did not affect the proportion of brood cells that contained pollen balls (*F* = 0.72; *df* = 1; *P* = 0.41), or other non-viable material (*F* = 4.07; *df* = 1; *P* = 0.06) (Table 3).

**Table 3:** The proportion (±SE) of brood cells produced by *Megachile rotundata* from which an adult emerged or that contained a dead larva, pollen ball, or other non-viable material.

	Alfalfa	Buckwheat	Purple Tansy^b^
Emerged adult	31.93 ± 6.26 a^a^	11.02 ± 3.31 b	29.52 ± 10.64
Dead larva	10.97 ± 2.96 b	48.70 ± 3.73 a	53.01 ± 10.31
Pollen ball	4.19 ± 1.21 a	5.61 ± 1.10 a	3.61 ± 5.40
Other non-viable material	52.9 ± 7.19 a	34.67 ± 5.40 a	13.85 ± 0.60

**Notes.**

a For alfalfa and buckwheat, proportions within a row followed by different letters are significantly different (*α* = 0.05).

b Data collected from purple tansy plots were not included in statistical analyses; see methods for details.

### Purple tansy

#### Nesting and foraging activity

In purple tansy plots, the number of active adults increased with temperature (*χ*^2^ = 18.3; *df* = 1; *P* < 0.005), and declined over time (*χ*^2^ = 8.21; *df* = 1; *P* = 0.004) (Fig. 2C). Similarly, the number of foraging trips increased with temperature (*χ*^2^ = 33.7; *df* = 1; *P* < 0.005) and decreased over the course of the experiment (*χ*^2^ = 21.4.6; *df* = 1; *P* < 0.005) (Fig. 3C). The number of resting females decreased with temperature (*χ*^2^ = 17.1; *df* = 1; *P* < 0.005) and was not affected by days after release (*χ*^2^ = 1.4; *df* = 1; *P* = 0.24), while the proportion of individuals observed at the nest box declined over the course of the experiment (*χ*^2^ = 52.2; *df* = 1; *P* < 0.005).

Finally, we did not observe females collecting or using purple tansy leaves for brood cell production. Instead, they collected leaf tissue from the few weeds present in the plots, primarily lamb’s quarters (*Chenopodium album*).

#### Reproduction and development

The mean number of brood cells produced per purple tansy plot was 41.50 ± 4.35 (Table 1). The proportion of these brood cells from which an adult emerged was 29.52 ±  10.64 (Table 3). Females produced from purple tansy plots weighed significantly more than males produced from purple tansy plots (*F* = 6.90; *df* = 1, 45; *P* = 0.0117) (Table 2). The proportion of brood cells that contained a dead larva, pollen ball, or non-viable material was 53.01 ± 10.64, 3.61 ± 5.40, and 13.85 ± 0.6, respectively (Table 3).

## Discussion

ALB foraging activity and development differed on buckwheat and alfalfa plots under our experimental conditions. At the high release rate, foraging activity was higher on buckwheat plots compared to both release rates on alfalfa plots, and the number of foraging trips by females increased over time on buckwheat plots. However, ALB development was poor on buckwheat overall: Although twice the number of brood cells were produced on buckwheat plots at the high release rate, a lower proportion of those cells developed successfully into adults, and adults that did emerge were smaller compared to alfalfa plots. Horne (1995a) also observed high larval mortality of ALB that foraged exclusively on buckwheat, and adults that did emerge were extremely small (0.0049 ± 0.4 g). Buckwheat is highly attractive to ALB (Horne, 1995a; Horne, 1995b); however, it produces poor-quality pollen. The minimum level of crude protein in pollen needed to optimally support adults and brood rearing for honey bees is 20% (Kleinschmidt & Kondos, 1976), but buckwheat pollen contains only 11% (Somerville, 2001). Furthermore, buckwheat flowers are open only for 6–7 h per day and may not produce nectar on humid and/or overcast days (Alekseyeva & Bureyko, 2000). Therefore, ALB was likely resource limited in terms of quality and quantity on the buckwheat plots in our study, resulting in poor offspring development. A lack of resources may also explain the comparatively high foraging activity we observed on buckwheat plots: Females may have foraged more to compensate for the low availability and quality of nectar and pollen.

We did not observe a difference in overall foraging activity, reproduction, or development on alfalfa plots between the low and high adult release rates, which indicates that on a per individual basis, ALB performed better on alfalfa at the low release rate. Furthermore, we observed a lower proportion of females at the nest box at the high release rate. Therefore, release rates higher than 10 females should be avoided on alfalfa plots of the size used in our study (ca. 12 m^2^).

The mean number of brood cells produced on alfalfa plots at the 10 female release rate was 42 over the 18 d observation period in our study. In a similar field study, 20 female ALB nesting on alfalfa plots three times the size of our plots produced between 40 and 90 brood cells total in only 9 d (Pitts-Singer & Barbour, 2017). However, females in our study were produced ca. 0.25 brood cells per day, comparable to the 0.22 to 0.5 brood cells produced per day in Pitts-Singer & Barbour (2017). This would suggest that ALB in our small plots were not limited by resources at the 10 female release rate.

In the first year of our study, purple tansy failed to germinate, and in the second year, its establishment was patchy. Conversely, for a 2015 study conducted in the same field, purple tansy established well when seeded at the same rate as in our study (Gradish et al., 2016), which indicates that the soil and climatic conditions at our experimental site are generally suitable for purple tansy. However, compared to 2015, the summers of 2016 and 2017 were hot and dry, particularly during our study period, and we believe that these conditions contributed to the poor growth of purple tansy during our study.

Regardless of the specific cause, because purple tansy failed to germinate in 2016, we could not statistically compare our results from purple tansy with those from alfalfa and buckwheat. In 2017, the patchy quality of our purple tansy stand limited the number of usable plots, and we were only able to examine a single adult release rate. Additionally, there were few days of suitable weather for observing ALB foraging and nesting activity. Although these limitations make it difficult to draw conclusions from our study about purple tansy, our general observations of ALB behavior and reproduction on purple tansy are important for informing further research into semi-field method development for ALB.

Our observations suggest that purple tansy should be investigated further as a potential surrogate plant for semi-field studies with ALB. In our study, ALB readily foraged and nested on purple tansy plots. ALB foraging and nesting activity on purple tansy overall was similar to that observed on alfalfa and buckwheat at the 10/20 release rate. Reproduction and development on purple tansy also was similar to the 10/20 release rate on alfalfa but higher compared to both release rates on buckwheat. Similarly, previous semi-field studies have found ALB reproduces well on larger plots of purple tansy at a release rate of 20♀/40♂ (Artz & Pitts-Singer, 2015). Interestingly, females in our study did not collect leaf pieces from purple tansy plants and instead used leaves from available weeds (primarily lamb’s quarters (*Chenopodium album*) but also some grasses). We were regularly removing weeds from our plots, which reduced the density of leaf tissue preferred by females and, in turn, may have negatively impacted brood cell production by females. Given our results and those from previous studies, further research into the reproduction and development of ALB on small plots of purple tansy is warranted. Purple tansy might be able to support a higher release rate of ALB if plots were supplemented with a suitable alternative source of leaf tissue. In particular, ALB is highly attracted to buckwheat leaf tissue (Horne, 1995a; Horne, 1995b), and perhaps a mixed planting of purple tansy and buckwheat may support ALB better than purple tansy alone.

Some of the endpoints we measured, such as the number of active adults and resting females, did not vary among surrogate plants and release rates. While these endpoints were not particularly informative in our study, they were relatively easy to measure and may be valuable in the context of pesticide risk assessment. For example, sub-lethal exposure to pesticides is known to reduce the activity of adult bees (Gill & Raine, 2014; Tome et al., 2015; Tosi & Nieh, 2017). For ALB, a reduction in activity could manifest as more individuals “resting” in the nest box or a reduction in the number of adults foraging or flying. While a reduction in activity could also be detected by counting the number of foraging trips, including counts of individuals performing different sets of behaviors would allow for a more complete account of what is occurring in the plot. Furthermore, the number of active adults also captures information about males, and while not the primary focus of pesticide risk assessments, data on male response to pesticides is valuable. For example, a difference in the number of active individuals between pesticide risk assessment treatments without a corresponding difference in foraging trips may indicate a difference in the susceptibility between males and females that wouldn’t be detected by counting number of foraging trips alone.

The plot size used in our experiment was selected to accommodate the commercially available enclosures described in the methods section. There are advantages to using these small enclosures: they are easy to set up, transport, and store; their small size lends itself to greater replication; and they are more economical compared to larger structures or field tunnels (Gradish et al., 2016). Our results from alfalfa suggest that ALB are not resource limited on the small plots, and therefore, we believe there is merit is using a small plot set-up for semi-field experiments with ALB.

In conclusion, our results suggest that buckwheat alone is not a suitable surrogate plant for use in semi-field studies with ALB. Despite being highly attractive to ALB (Horne, 1995a; Horne, 1995b), our results are similar to previous observations that buckwheat does not adequately support ALB reproduction and development as well as alfalfa. In contrast, alfalfa appears to support ALB on small plots. For alfalfa, we recommend a maximum release rate of 10♀/20♂ in 12 m^2^ plots. ALB also performed well on purple tansy, and our observations of ALB behaviour and reproduction on purple tansy under semi-field conditions suggest that further research into the use of this surrogate plant is warranted. However, females did not use purple tansy leaf tissue to construct brood cells. Since an alternative source of leaf tissue is required for ALB to nest in purple tansy, we suggest that future semi-field studies examine supplementing purple tansy plots with another plant with suitable leaf tissue, such as buckwheat, as it may enhance ALB nesting success and reproduction.

##  Supplemental Information

10.7717/peerj.6278/supp-1 Table S1 Observation schedule for the semi-field experiments conducted in 2016 and 2017 † Only one set of observations was collected due to thunder storms.
‡ No observations collected due to adverse weather.

10.7717/peerj.6278/supp-2 Data S1 Nesting and Foraging Activity Data

10.7717/peerj.6278/supp-3 Data S2 Reprodcution and Development data

## Additional Information and Declarations

### Data Availability

The following information was supplied regarding data availability: Raw data is available in the Supplementary Information.
