# Supplementary material for: Potential surrogate plants for use in semi-field pesticide risk assessment with Megachile rotundata
Source: PeerJ. 2019 Jan 18;6:e6278. doi: 10.7717/peerj.6278 (PMC6340348; doi:10.7717/peerj.6278)
Supplement: Table S1 — † Only one set of observations was collected due to thunder storms. ‡ No observations collected due to adverse weather. [file peerj-07-6278-s001.docx]

**Supplementary Table 1.**

Observation schedule for the semi-field experiments conducted in 2016 and 2017.

| **Activity** | **Buckwheat 2016** | **Alfalfa 2016** | **Purple Tansy 2017** |
| --- | --- | --- | --- |
| Seeding | May 19 | NA | June 19 |
| Enclosures Installed | July 4 | July 27 | Aug 11 |
| ALB Released | July 5 | July 28 | Aug 13 |
| Observations | July 6 | July 29 | August 14 |
|  | July 8 | August 1 † | August 17‡ |
|  | July 11 | August 3 | August 18 |
|  | July 13 | August 5 | August 20 |
|  | July 15 | August 8 | August 23 |
|  | July 18 | August 10 |  |
|  | July 20 | August 12 ‡ |  |
|  |  | August 15 |  |

† Only one set of observations was collected due to thunder storms

‡ No observations collected due to adverse weather
